# The dynamics of GII.4 Norovirus in Ho Chi Minh City, Vietnam^☆^

**DOI:** 10.1016/j.meegid.2013.04.014

**Published:** 2013-08

**Authors:** Phan Vu Tra My, Ha Minh Lam, Corinne N. Thompson, Hoang Le Phuc, Pham Thi Ngoc Tuyet, Ha Vinh, Nguyen Van Minh Hoang, PhamVan Minh, Nguyen Thanh Vinh, Cao Thu Thuy, Tran Thi Thu Nga, Nguyen Thi Thu Hau, Nguyen Tran Chinh, Tang Chi Thuong, Ha Manh Tuan, James I. Campbell, Archie C.A. Clements, Jeremy Farrar, Maciej F. Boni, Stephen Baker

**Affiliations:** a Wellcome Trust Major Overseas Programme, Oxford University Clinical Research Unit, 764 Vo Van Kiet, District 5, Ho Chi Minh City, Viet Nam; b Centre for Tropical Medicine, Nuffield Department of Clinical Medicine, University of Oxford, Wellington Square, Oxford OX1 2JD, United Kingdom; c Children’s Hospital 1, 341 Su Van Hanh, District 5, Ho Chi Minh City, Viet Nam; d Children’s Hospital 2, 14 Ly Tu Trong, District 1, Ho Chi Minh City, Viet Nam; e Hospital for Tropical Diseases, 764 Vo Van Kiet, District 5, Ho Chi Minh City, Viet Nam; f University of Queensland, School of Population Health, Brisbane St Lucia, QLD 4072, Australia; g The London School of Hygiene and Tropical Medicine, Keppel Street, London WC1E 7HT, United Kingdom

**Keywords:** Norovirus, GII.4, Phylogenetic, Spatial, Temporal, Cluster

## Abstract

Norovirus (NoV) is a major cause of epidemic gastroenteritis in industrialized countries, yet the epidemiological significance of NoV in industrializing countries remains poorly understood. The spatiotemporal distribution of NoV genotypes identified in 2054 enrolled children was investigated between May 2009 and December 2010, in Ho Chi Minh City (HCMC), Vietnam. A total of 315 NoV extracted from stool samples were genotyped and GPS mapped to their source. Genogroup II NoV, particularly GII.4, were predominant, and the GII.4 strains could be subgrouped into GII.4-2006b (Minerva) and GII.4-2010 (New Orleans) variants. There was no spatiotemporal structure among the endemic GII strains; yet a significant spatiotemporal signal corresponding with the novel introduction of GII.4-2010 variant was detected. These data show that NoV GII.4 variants are highly endemic in HCMC and describe a scenario of rapid NoV strain replacement occurring in HCMC in early 2010.

## Highlights

• NoV was identified in the stools of diarrheal patients and controls in HCMC.
• The locations of the NoV infections were GPS mapped.
• A novel NoV GII.4-2010 (New Orleans) variant was detected.
• The NoV GII.4-2010 demonstrated a significant spatiotemporal signal.

## 1 Introduction

Norovirus (NoV) is a non-enveloped positive-sense single-stranded RNA virus belonging to the taxonomic family *Caliciviridae* (Green et al., 2000; Jiang et al., 1990). NoV accounts for a significant proportion of the global burden of viral gastroenteritis (Glass et al., 2000, 2009; Patel et al., 2009, 2008), and up to 50% of all-cause outbreaks of diarrhea (Patel et al., 2009). The disease typically presents as acute watery diarrhea with vomiting and a low-grade fever (Patel et al., 2009), and is usually self-limiting, lasting between one and three days, but can be aggressive, severe and protracted in young children, the elderly and the immuno-compromised (Estes et al., 2006). NoV has an exceptionally low infectious dose (10–100 viral particles (Patel et al., 2009)) and can survive on surfaces for prolonged time periods (Weber et al., 2010); as a result, NoV frequently causes explosive gastroenteritis epidemics (Estes et al., 2006; Patel et al., 2009, 2008).

The 7.5 kb genome of NoV has three open reading frames (ORFs), encoding the RNase-dependent RNA polymerase (RdRp, ORF1), the major capsid protein (VP1, ORF2) and the minor capsid protein (VP2, ORF3) (Bertolotti-Ciarlet et al., 2003; Jiang et al., 1993). The current classification divides NoV into five genogroups (GI – GV) on the basis of sequence identity within the major capsid protein (VP1). Genogroups GI, GII and GIV are associated with infections in humans (Zheng et al., 2006). Molecular characterization of coding sequences within RdRp (region A and B) (Ando et al., 1995, 2000; Fankhauser et al., 2002; Jiang et al., 1999; Vinje and Koopmans, 1996) and ORF2 (region C, D and E) (Kageyama et al., 2004; Kojima et al., 2002; Noel et al., 1997; Vinje et al., 2004) is targeted for NoV detection, genogrouping and genotyping. Genogroups I and II are the most common cause of human infections (Donaldson et al., 2010), and can be differentiated into 8 GI and 23 GII capsid genotypes, and 14 GI and 29 GII polymerase genotypes (Kroneman et al., 2011; Zheng et al., 2006).

The epidemiology of NoV is complex and is influenced by a multitude of factors, including population immunity, the environment, and seasonality (Donaldson et al., 2010; Marshall and Bruggink, 2011), making molecular epidemiology challenging. The acknowledged interpretation of global NoV epidemiology, particularly GII.4 genotype, is that strain replacement occurs every two to three years (Bull et al., 2010; Bull and White, 2011; CDC, 2010; Donaldson et al., 2008; Siebenga et al., 2009). Over the last two decades, these replacements were typically caused by strains of a single lineage of a GII.4 genotype, which have been responsible for the majority of NoV outbreaks worldwide since being first identified in the USA in the mid 1990s (Bull and White, 2011; Noel et al., 1999). At least four major global NoV replacements have been described since 1995, each due to a novel GII.4 variant (Bull et al., 2006; Noel et al., 1999; Siebenga et al., 2010; Tu et al., 2008), believed to have escaped immunity in the population through antigenic variation (Lindesmith et al., 2012b, 2012c, 2011).

The majority of NoV studies are performed in industrialized countries and disease outbreaks are continually monitored through several disease surveillance networks (Vega et al., 2011; Verhoef et al., 2009). However, little is known about the transmission, molecular diversity or spatiotemporal dynamics of NoV infections in areas with differing public health infrastructure and demographics. Vietnam is an industrializing country with densely populated urban centers and a changing spectrum of infectious diseases as a presumed consequence of rapid economic development and urbanization (Vinh et al., 2009). NoV was first reported in Ho Chi Minh City (HCMC) in 1999 (Hansman et al., 2004), and our recent work has demonstrated that NoV is endemic throughout the year, in contrast to the winter outbreaks observed in temperate locations (Lopman et al., 2009; Mounts et al., 2000). To understand the epidemiology and NoV strain diversity in HCMC, we investigated the molecular and spatiotemporal distribution of NoV genotypes in young hospitalized children between May 2009 and December 2010 in HCMC, Vietnam.

## 2 Material and methods

### 2.1 Study setting and design

This study was conducted according to the principles expressed in the Declaration of Helsinki and was approved by the ethical review boards of Children’s Hospital 1 (HCMC), Children’s Hospital 2 (HCMC), the Hospital for Tropical Diseases (HCMC), and the Oxford Tropical Research Ethics Committee (OxTREC Approval No. 0109) (Oxford). The parents or legal guardians of the enrolled children were required to provide written informed consent for sample collection and residential mapping.

A stool specimen was collected within 24 h of enrollment from each recruited individual (1443 diarrheal patients and 611 asymptomatic controls). These participants (*N* = 2054) were children of 0–60 months of age and residents of HCMC, Vietnam, over the study period from May 2009 to December 2010. Diarrheal patients were children with acute diarrheal disease (⩾3 loose stools or at least one bloody loose stool within 24 h period (WHO, 2005)) who were admitted to the three study sites and had not received treatment with antimicrobials in the three days prior to hospital admission. Asymptomatic controls were diarrhea-free children attending Children’s Hospital 1 or Children’s Hospital 2 for nutritional health checks or for other gastrointestinal issues unrelated to diarrhea or gastroenteritis without any history of diarrhea, respiratory illness or treatment with antimicrobials within 7 days of study enrollment.

### 2.2 Norovirus detection

Total viral RNA was extracted and reverse transcribed into cDNA as previously described (Tra My et al., 2011). Norovirus genogroup I (GI) and II (GII) were detected in separate reactions by conventional Reverse Transcriptase Polymerase Chain Reaction (RT PCR) using consensus primers, G1SKF/G1SKR (Kojima et al., 2002) and COG2F/G2SKR (Kageyama et al., 2003; Kojima et al., 2002) for GI and GII, respectively. These PCR primers amplify a region between position 5342 and 5671 (330 bp) in the genome of NoV GI (Norwalk/68, GenBank accession No. M87661) containing an overlap of 17 bp of 3′ end ORF1 and 313 bp of 5′ end ORF2, and between position 5003 and 5389 (387 bp) in the genome of NoV GII (Lordsdale/93, GenBank accession No. X86557) containing an overlap of 83 bp of 3′ end ORF1 and 304 bp of 5′ end ORF2.

### 2.3 Norovirus genotyping

NoV positive PCR amplicons were purified using the QIAquick PCR purification kit (QIAGEN, Hilden, Germany), and subjected to direct sequencing using the amplification primers. DNA concentrations were determined using a NanoDrop ND-1000 spectrophotometer (Thermo Fisher Scientific, United Kingdom) and direct sequencing was performed using a BigDye Terminator Cycle Sequencing kit (Applied Biosystems, USA) and generated with an ABI Prism 3130xl Genetic Analyzer (Applied Biosystems, USA). DNA sequences were assembled using DNA Baser Sequence Assembler v3.0.17 (Heracle Biosoft, Pitesti, Romania). NoV genotypes were assigned based on ORF2 sequences using the online Norovirus Automated Genotyping Tool, as directed (Kroneman et al., 2011).

### 2.4 Construction of NoV phylogenies

DNA sequences were uploaded into GenBank (HE716437 to HE716751) and used for local phylogenetic construction. Manual alignment of all sequences was performed in Se-AL (http://tree.bio.ed.ac.uk/software/figtree/) prior to phylogenetic reconstruction. Maximum likelihood (ML) trees were inferred using RAxML (Stamatakis et al., 2008), employing the general-time reversible model of nucleotide substitution with a gamma distribution of among-site rate variation (GTR + Γ) and 1000 bootstrap replicates. Resulting trees were visualized in FigTree v1.3.1 (http://tree.bio.ed.ac.uk/software/figtree/) and mean pairwise genetic distances were estimated in MEGA 5 (Tamura et al., 2011).

Two hundred and sixty-nine global GII.4 strains encompassing the global diversity of GII.4 variants were retrieved from GenBank (http://www.ncbi.nlm.nih.gov/); 43 of these strains originated from previous studies conducted in Vietnam (Hansman et al., 2004; Nguyen et al., 2008, 2007a, 2007b; Tamura et al., 2010; Trang et al., 2012). In addition, available archived samples that were Enzyme-Immuno Assay (EIA) positive for NoV (GI and GII) from our previous work in 2008 from southern Vietnam (Tra My et al., 2011) were also selected for analysis and genotyped using the same methodology.

The time of isolation for each of the NoV strains was retrieved from GenBank or the publication associated with the sequence, and the year of isolation was used to calculate evolutionary rate. All sequences were aligned in Se-AL and trimmed to 378 bp to correspond with the sequences identified in this study to maximize sequence homology for phylogenetic reconstruction. Phylogenetic reconstructions of relationships among the GII.4 variants identified in this study and global GII.4 sequences were inferred using the Bayesian Markov chain Monte Carlo (MCMC) method as implemented in BEAST (Drummond and Rambaut, 2007). A GTR substitution model with gamma-distributed rate variation and a relaxed uncorrelated lognormal clock model with a constant population size were employed. The MCMC analysis was run for 50 million generations (with a burn-in of 5 million) and analyzed using Tracer (http://tree.bio.ed.ac.uk/software/tracer/) to ensure that all parameters had converged. Maximum clade credibility trees were annotated using TreeAnnotator v1.6.1 (BEAST) and visualized in FigTree v1.3.1. The weighted average evolutionary rates across branches were assessed in Tracer.

### 2.5 Mapping of corresponding residential addresses

The location of each enrollee’s residence was recorded using an eTrex Legend GPS device (Garmin, United Kingdom) and verified by an additional member of the study team. Latitude and longitude of each residence (recorded in decimal degrees) were entered along with patient metadata in Microsoft Excel (Microsoft, Redmond, USA). Location data were converted to KML format and locations were visualized and validated in Google Earth version 5 (http://www.google.com/earth/index.html) (Supplementary Figure).

### 2.6 Spatiotemporal analyses

Mantel tests were performed to assess potential correlations between genetic, temporal, and spatial distances of GII strains and variants within the GII.4 clade, using the *ade4* package in R (R_Development_Core_Team, 2011) (ww.ats.ucla.edu/stat/r/faq/mantal_test.htm). A Bernoulli model was used to examine spatiotemporal clusters of GII.4-2010, using all non-GII.4-2010 to represent the background distribution of the NoV population using SaTScan v9.1.1 software (http://www.satscan.org/). For the current analysis, the upper limit for cluster detection was specified as 10% of the study population over 10% of the study duration. The significance of the detected clusters was assessed by a likelihood ratio test, with a *p*-value obtained by 999 Monte Carlo simulations generated under the null hypothesis of a random spatiotemporal distribution.

## 3 Results

### 3.1 The temporal distribution of NoV genogroups and genotypes

Over the period of study, 315 NoV positive stool samples from 2054 individuals were identified (15.3%). Genotyping of these 315 strains demonstrated that the predominant NoV genogroup was GII (304; 96.5%), with NoV GI identified in only 3.5% of enrollees (Table 1). An array of GI (GI.3, GI.4 and GI.5) and GII (GII.2, GII.3, GII.4, GII.6, GII.7, GII.9, GII.12, GII.13 and GII.4U (genotype 4 with unassigned variants, outgroup of the lineage containing strains OB200615 and X76716 according to RIVM-NoroNet)) genotypes were additionally detected. Among the 304 NoV GII strains, GII.4 was the most frequently identified (247; 81.3%) and could be separated into two major variants, GII.4-2006b (Minerva) (86.2%; 213/247) and GII.4-2010 (New Orleans) (12.1%; 30/247). The GII.4-2006b and GII.4-2010 variants were the focus of subsequent analyses as a consequence of their overall dominance and their perceived epidemiological relevance in HCMC.

**Fig. 1:**
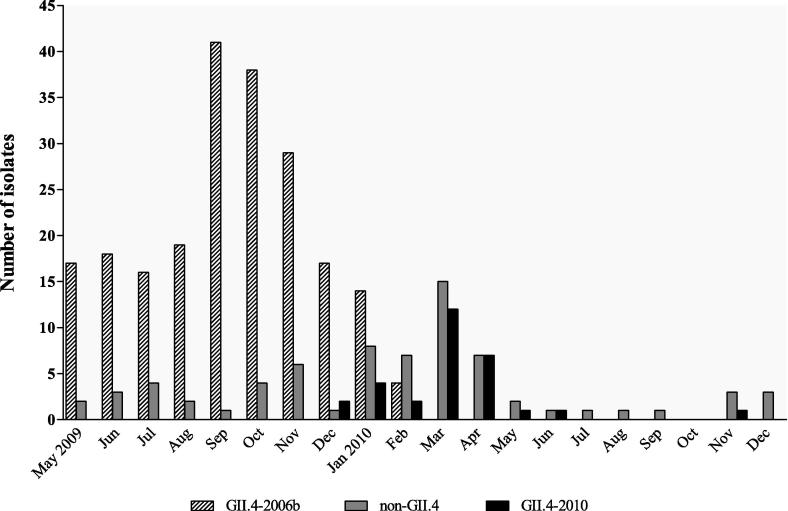
The temporal distribution of NoV GII.4 variants in HCMC over the period of study, from May 2009 to December 2010. Graph showing the distribution of GII.4-2006b and GII.4-2010 variants against other NoV strains (total numbers identified) detected in symptomatic and asymptomatic children over the study period (GenBank accession number HE716437 to HE716751).

The genotyping data were combined with isolation dates to illustrate the distribution of GII.4 variants over the period of sample collection (Fig. 1). NoV GII.4 strains were detected every month from May 2009 to April 2010, with GII.4-2006b being the sole GII.4 variant identified between May and November 2009. However, this trend was not uniform as there was a substantial increase in GII.4-2006b infections during September and October 2009. The GII.4-2010 variant was first detected in December 2009 and became increasingly prevalent in the following months. Concurrently, the proportion of GII.4-2006b variants decreased from 7.36% (17/231) in December 2009 to 1.73% (4/231) in February 2010, and was not detected after March 2010. However, trends in the distribution of NoV variants were difficult to assess during the period after April 2010 due to the limited number of enrollees recruited in this period.

### 3.2 Phylogenetic analyses of NoV sequences

**Fig. 2:**
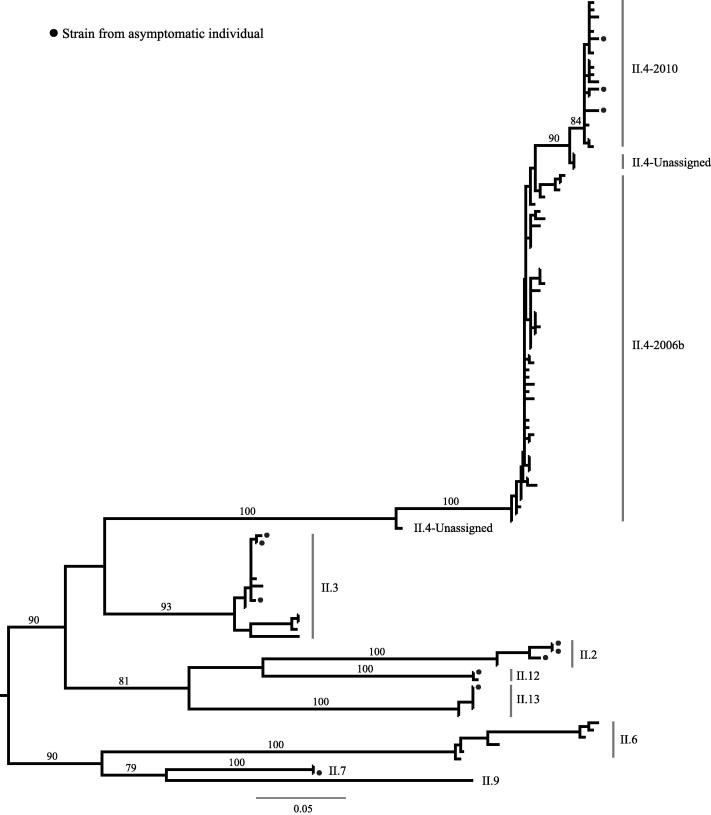
Phylogenetic tree of 109 NoV GII strains from HCMC. Tree constructed from 109 NoV GII strains collected during this study and based on the GII amplification fragment trimmed to 378 bp. All horizontal branch lengths are drawn to the scale of a nucleotide substitution per site. Tree is mid-point rooted with branches according to viral genotypes/variants. Strains from asymptomatic individuals are labeled with a circle. Only bootstrap values of >75 are shown.

Phylogenetic analyses were performed on all GI and GII NoV sequences, and the mean uncorrected genetic distances among the strains within the GII and between variants within the GII.4 genotype (pairwise distance of maximum composite likelihood calculation) were 0.147 and 0.016 substitutions/site, respectively. Based on this primary phylogenetic analysis, the GI strains were excluded and the GII strains were subsampled by removing identical GII sequences to reconstruct a maximum likelihood phylogenetic tree summarizing the genetic diversity present in HCMC (*N* = 109) (Fig. 2).

**Fig. 3:**
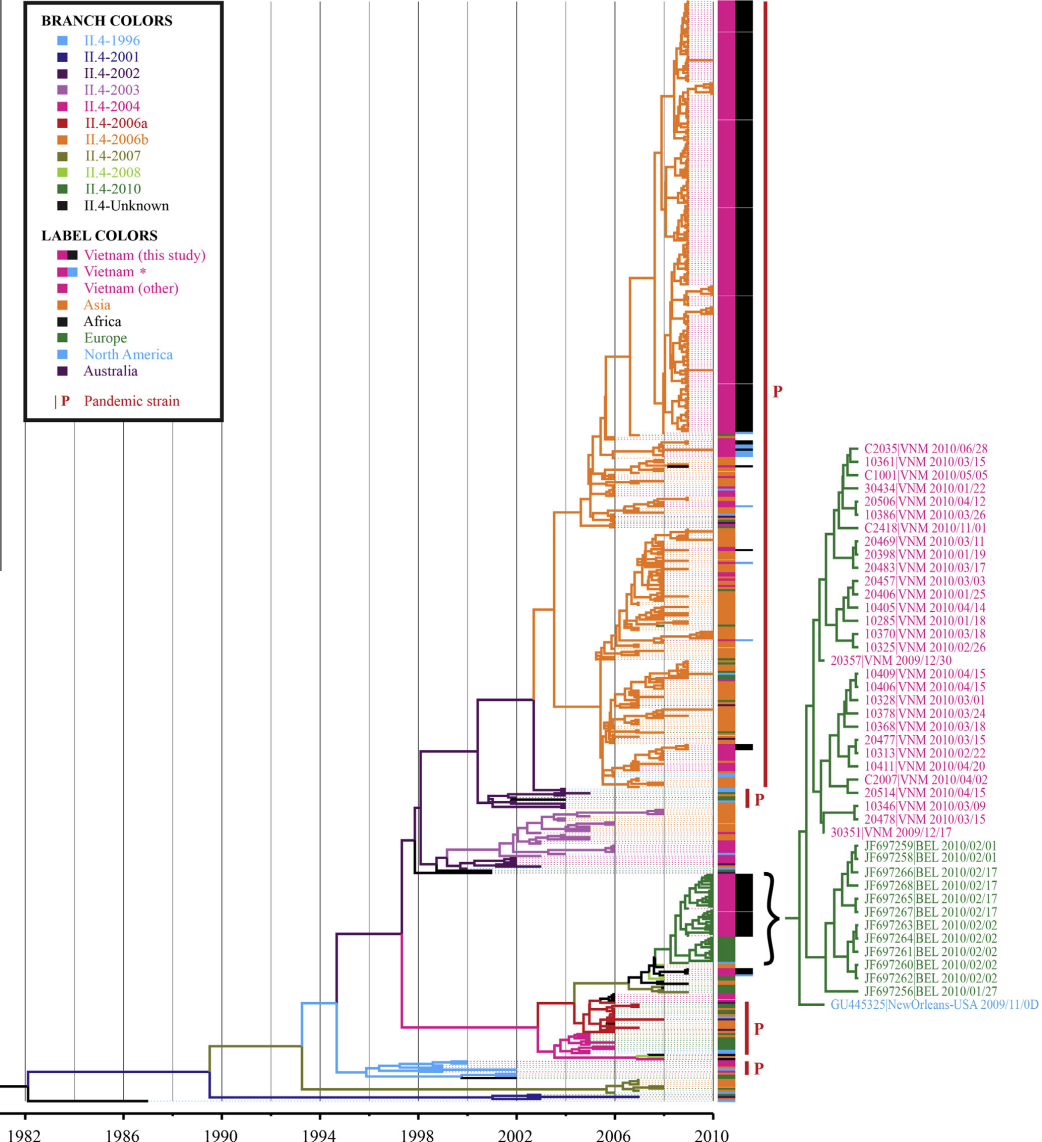
Phylogenetic tree of GII.4 NoV strains from HCMC and globally representative sequences. Maximum likelihood phylogenetic tree of 526 global and HCMC GII.4 NoV strains constructed from the amplification region trimmed to 378 bp. All horizontal branch lengths are drawn to the scale of a nucleotide substitution per site per year. Branch tips are colored according to the viral genotype and color-coded by their continent of isolation. Vietnamese strains included are from this study (*N* = 247), other studies (“other”; *N* = 43) and from a 2008 study in southern Vietnam (*N* = 10) (Tra My et al., 2011) denoted by (∗). The GII.4-2010 clade is magnified to highlight the strains originating from Vietnam, Belgium and America.

Sequences of the two GII.4 variants from HCMC (*N* = 247) were compared with 269 global sequences and 10 selected GII.4 sequences isolated in 2008 in southern Vietnam (Tra My et al., 2011) (Fig. 3). Using the Bayesian MCMC method and time-stamped sequences, the evolutionary rate of NoV GII.4 was estimated to be 8.072 × 10^−3^ substitutions/site/year (95% Highest Probability Density (HPD): 6.195 × 10^−3^, 1.012 × 10^−2^). The GII.4-2006b sequences from NoV originating in Vietnam fell in the same clade as global GII.4-2006b viruses, with clustering unrelated to the time or place of isolation. This GII.4-2006b lineage could be further divided into two sub-lineages; strains from HCMC could be found in both, confirming co-circulation of divergent GII.4-2006b viruses. Notably, the upper sub-lineage contained more sequences from this study while more Vietnamese strains from previous studies fell in the lower sub-lineage. The GII.4-2010 strains clustered in a single lineage, separate from the GII.4-2006b lineage. The GII.4-2010 lineage could be differentiated partially by location, with Vietnamese and Belgian sub-lineages stemming from the New Orleans GII.4-2010 variant.

### 3.3 Spatiotemporal clustering of NoV in HCMC

**Fig. 4:**
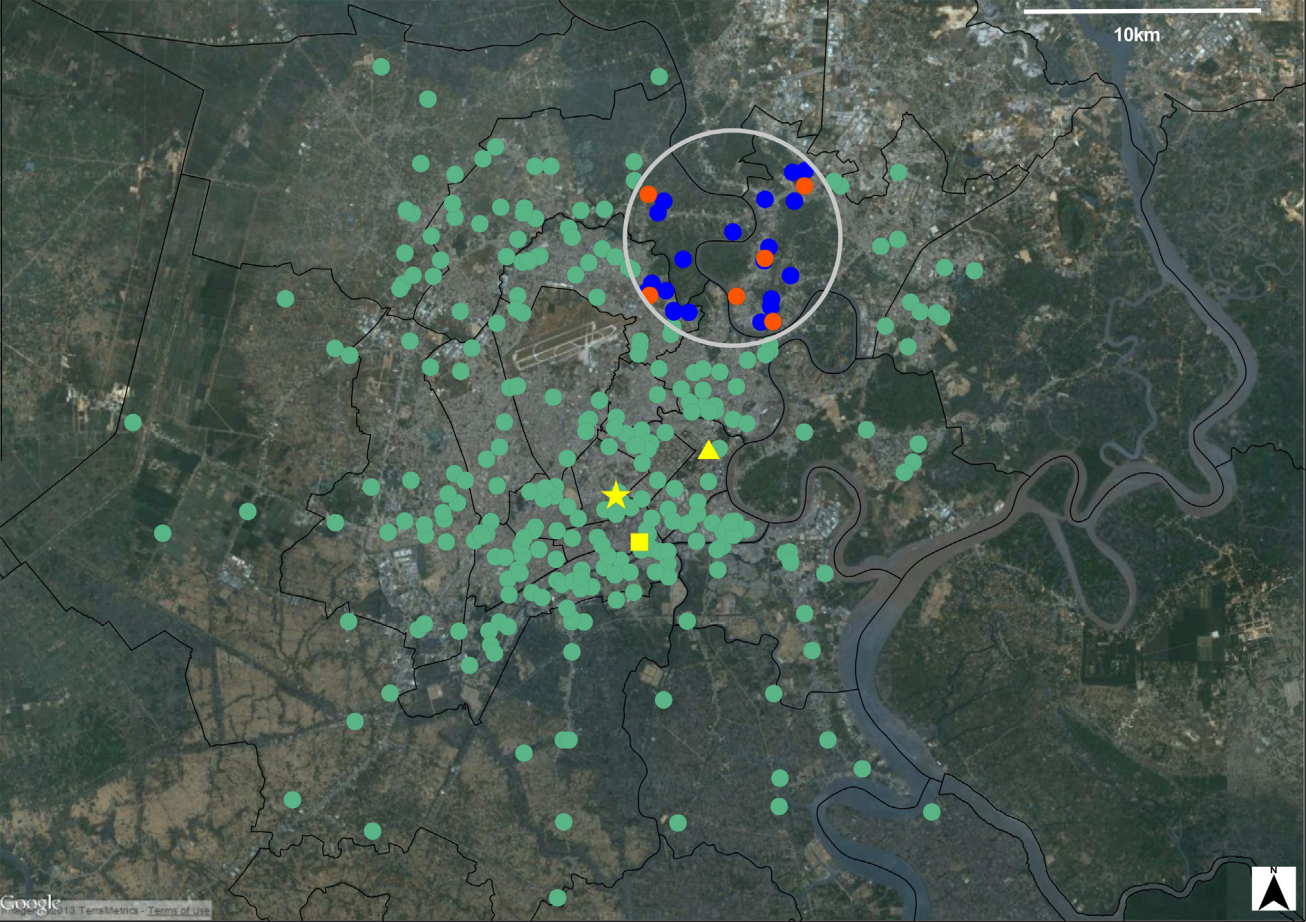
The spatiotemporal clustering of NoV GII.4-2010 strains. When compared to all other GII strains, GII.4-2010 strains were found to cluster in the northeast of Ho Chi Minh City during March–April 2010. This significant cluster with radius 3.8 km, shown by the black circle, contained 6 GII.4-2010 NoV (0.59 expected, relative risk = 12.65, *p* = 0.0003). Orange dots represent NoV GII.4-2010 strains located within the cluster, blue dots represent the non-GII.4-2010 NoV within the radius of the cluster, and the green dots represent all other NoV strains (GII.4-2010 and non-GII.4-2010) found over the study period that were not found to cluster. The yellow square indicates the location of the Hospital for Tropical Diseases, the star indicates Children’s Hospital 1 and the triangle indicates Children’s Hospital 2. The thin black lines represent district boundaries within Ho Chi Minh City.

The temporal data suggested that a NoV strain replacement occurred during the period of investigation. There was a significant association between the genetic distance of strains within GII and their date of isolation (*p* < 0.0001; Mantel test), this association was particularly apparent between the GII.4 sequences and their isolation date (*p* < 0.0001; Mantel test). However, there was no similar association between geographical distance and genetic distance (*p* = 0.197 for GII strains; *p* = 0.844 for GII.4 sequences), or between isolation date and geographical distance (*p* = 0.248 for GII strains; *p* = 0.851 for GII.4 sequences). These data indicate a lack of a local transmission signal of NoV in HCMC. Yet, a spatiotemporal cluster detection analysis performed in SaTScan supported our original hypothesis, detecting a cluster of six GII.4-2010 NoV (over other NoV GIIs (0.59 expected)) in a 3.8 km radius in the northeast of the City (relative risk = 12.65, *p* = 0.0003) (Fig. 4), indicating that the initial dynamics of GII.4-2010 were highly localized during their introduction period into HCMC.

## 4 Discussion

There are inadequate data regarding the burden of NoV disease in industrializing countries such as Vietnam; this limits our knowledge of viral distribution, transmission chains and local microevolution. Data on NoV genotype distribution across a range of geographical locations through time is essential for understanding global NoV epidemiology. This is particularly important with respect to the ongoing development and clinical trials of NoV vaccines (Atmar et al., 2011; El-Kamary et al., 2010; Parra et al., 2012), which should be developed in consideration of global and regional strain circulation and their ability to induce cross-protection. Here, by examining the genetic, spatial and temporal dynamics of NoV in children in HCMC, we aimed to assess the local molecular epidemiology of NoV. Our data show a diverse array of NoV genotypes and the emergence of a novel variant. The emergence of the GII.4-2010 and subsequent lack of GII.4-2006b isolates suggest that a rapid strain replacement event may have occurred in the population, although the small numbers of isolates from the latter half of the study preclude strong inference on these dynamics.

Strains belonging to GII are responsible for the vast proportion of human NoV infections worldwide, and GII.4 variants play a particularly important role in pediatric NoV infections (Bull and White, 2011; Lopman et al., 2004; Siebenga et al., 2009). Here, a variety of NoV GII genotypes were found to be co-circulating, with GII.4 predominating. This observation is consistent with work originating in northern Vietnam (Trang et al., 2012) and other locations across Asia (Zeng et al., 2012), and we confirm that GII.4-2006b has continued to circulate in southern Vietnam since it was first detected in 2005 (Nguyen et al., 2008).

The GII.4-2010 variant detected here in December 2009, and first identified in October 2009 in New Orleans (USA) (Vega et al., 2011), was also reported in Belgium (Mathijs et al., 2011) and then internationally (Greening et al., 2012; McAllister et al., 2012; Nguyen and Middaugh, 2012; Puustinen et al., 2011; White et al., 2012), suggesting that this variant is the first globally disseminated strain to emerge since the pandemic GII.4-2006b (Minerva). The phylogenetic analyses demonstrated that the GII.4-2010 strains from the USA, Belgium and Vietnam were closely related, suggesting that these strains may have been introduced into Vietnam from the USA or Europe in 2009. Furthermore, the substitution rate for GII.4 (8.072 × 10^−3^ substitutions/site/year) estimated here is higher than previously reported (between 3.9 × 10^−3^ and to 5.3 × 10^−3^ substitutions/site/year) (Bok et al., 2009; Bull et al., 2010; Siebenga et al., 2010). This new estimate might reflect an increase in the rate of GII.4 evolution involving GII.4-2010 viruses, since Bok et al. (2009), Bull et al. (2010) and Siebenga et al. (2010) would have used a different sequence dataset (i.e. no GII.4-2010 sequences) given that their work was conducted prior to the emergence of GII.4-2010 (Bok et al., 2009; Bull et al., 2010; Siebenga et al., 2010). However, it is important to note that differences in the region of sequence selected for analysis (partial 5′ capsid herein versus complete capsid in previous studies), in addition to the differential method of evolutionary inference (linear regression, strict or relaxed clock, uncorrelated lognormal or exponential model) or the measured unit of time, do not enable an accurate evolutionary comparison between studies. Nevertheless, the phenomenon observed in this study certainly warrants further investigation.

A spatiotemporal signal for GII.4-2010 was detected for several months after it was introduced into HCMC but there was no similar spatiotemporal association for GII.4-2006b and non-GII.4. A potential explanation for the absence (GII.4-2006b and non-GII.4) and presence (GII.4-2010) of spatial signals in the NoV sequences is that GII.4-2006b and non-GII.4 genotypes were in a state of equilibrium when GII.4-2010 was introduced, and the GII.4-2010 exhibited an outbreak dynamic and exponential growth upon introduction. Similar replacement of GII.4-2006b viruses following the introduction of GII.4-2010 has been observed in Belgium (Mathijs et al., 2011), and NoV strain replacement has been observed in various NoV pandemics. These replacements include, GII.4-1997 (USA 95/96 variant), -2002 (Farmington Hills variant), -2004 (Hunter variant), -2006 comprising of GII.4-2006a (Laurens variant) and the -2006b (Minerva variant) (Bull and White, 2011; Donaldson et al., 2008; Lindesmith et al., 2011). Novel pandemic NoV GII.4 strains emerge every two to three years and it appears that novel GII.4 genotypes are capable of replacing existing GII.4 variants but not other endemic strains (such as GII.3, GII.6, GII.2) (Bull et al., 2010; Bull and White, 2011; CDC, 2010; Donaldson et al., 2008; Lindesmith et al., 2011; Siebenga et al., 2009). However, there is no current consensus on the underlying mechanism of GII.4 strain emergence and replacement, but may be induced by antigenic drift and herd immunity escape (Debbink et al., 2012; Donaldson et al., 2010; Lindesmith et al., 2012a, 2012c). Recent studies on mapping blockade epitopes in GII.4 VLPs (virus-like particles) have provided evidence that variation in the major neutralizing epitopes facilitate evasion of herd immunity against GII.4-2006b (Minerva), thus facilitating the emergence of GII.4-2010 (New Orleans) (Lindesmith et al., 2012b, 2012c, 2011).

Our study has some limitations; including not tracking the source and/or route of transmission or examining the genotype distribution after the period of investigation. Therefore, it is difficult to determine if the shift in the distribution of the GII.4 variants over time is due to an emergent virus becoming fixed in the population, or a local outbreak in the northeast of the city. The short temporal investigation also limits the determination of the magnitude to which the local NoV dynamics observed in HCMC reflect or follow the global evolutionary trend, such that we are unable to determine whether GII.4-2010 viruses continued circulating within this setting after the study period or were capable of diffusing across the country in the presence or absence of GII.4-2006b viruses. The status of population-level immunity to NoV in the population of HCMC is unknown, so we are unsure if exposure to GII.4-2006b NoV is protective against the -2010 variants. These findings highlight a broader scientific issue concerning outstanding questions on immune cross-protection in the space of NoV variants (Lindesmith et al., 2012c, 2011). Furthermore, the analysis was not performed on whole genome sequences and focused on a fragment of the genome, which may restrict the phylogenetic interpretation. Whole genome sequencing would greatly improve the utility of NoV epidemiological datasets, specifically to study the evolution of novel GII.4-2010 variants, aiding the detection of genomic sites that may induce potential antigenic variation. Finally, our hospital-based study design may be influenced by healthcare-seeking behavior, and may not be representative of the NoV in the local community.

## 5 Conclusions

This study expands the knowledge of NoV in industrializing countries, outlining a range of endemic NoV genotypes over a one-year period in HCMC. The analysis describes the co-circulation of heterogeneous NoV strains, and reports the identification of GII.4-2010 (New Orleans) in Asia, resulting in the replacement of a GII.4-2006b variant by the emergent GII.4-2010 strain. In conclusion, during the period of study, NoV GII-4 infections in HCMC demonstrated a spatiotemporal phylogenetic relationship, driven by the emergence of the GII.4-2010 (New Orleans) variant.

## Financial support

This work was supported through funding from The Wellcome Trust Major Overseas Programme core funding and Vizions initiative [089276/B/09/Z], the Li Ka Shing Foundation – Univeristy of Oxford Global Health Programme [LG05], and in part by the International Society for Infectious Diseases (USA). PVTM is funded by a PhD fellowship from The Wellcome Trust Major Overseas Programme (UK) and the International Society for Infectious Diseases (USA). ACAC is funded by an Australian National Health and Medical Research Council Career Development Award (Grant No. 631619). SB is a Sir Henry Dale Fellow, supported by the Royal Society and the Wellcome Trust, UK [SHDF34523].

## Conflict of interest

The authors do not have a commercial or other association that might pose a conflict of interest. The funding bodies have no role in the study design, data collection and analysis, and the decision to publish. The authors wish to declare no competing interests.

## Figures and Tables

**Table 1:** The distribution of NoV genogroups and genotypes identified in enrollees over the sample collection period in HCMC, Vietnam.

Genogroup/Genotype	Total *N* (%)
**GI**	**11 (3.5)**
I.3	7 (2.2)
I.4	1 (0.3)
I.5	3 (1.0)
**GII**	**304 (96.5)**
II.2	4 (1.3)
II.3	32 (10.2)
II.4	247 (78.4)
*II.4-2006b*	*213* (*67.6*)
*II.4-2010*	*30 (9.5*)
*II.4U*	*4* (*1.3*)
II.6	8 (2.5)
II.7	3 (1.0)
II.9	1 (0.3)
II.12	2 (0.6)
II.13	7 (2.2)
Total	315

## Appendix A Supplementary data

### .Google Map

The following KML file contains the Google map of the most important areas described in this article.

Map KML file containing the Google map of the most important areas described in this article.
