# Supplementary figures and images for: The dynamics of GII.4 Norovirus in Ho Chi Minh City, Vietnam
Source: Infect Genet Evol. 2013 Aug;18:335–43. doi: 10.1016/j.meegid.2013.04.014 (PMC4047827; doi:10.1016/j.meegid.2013.04.014)

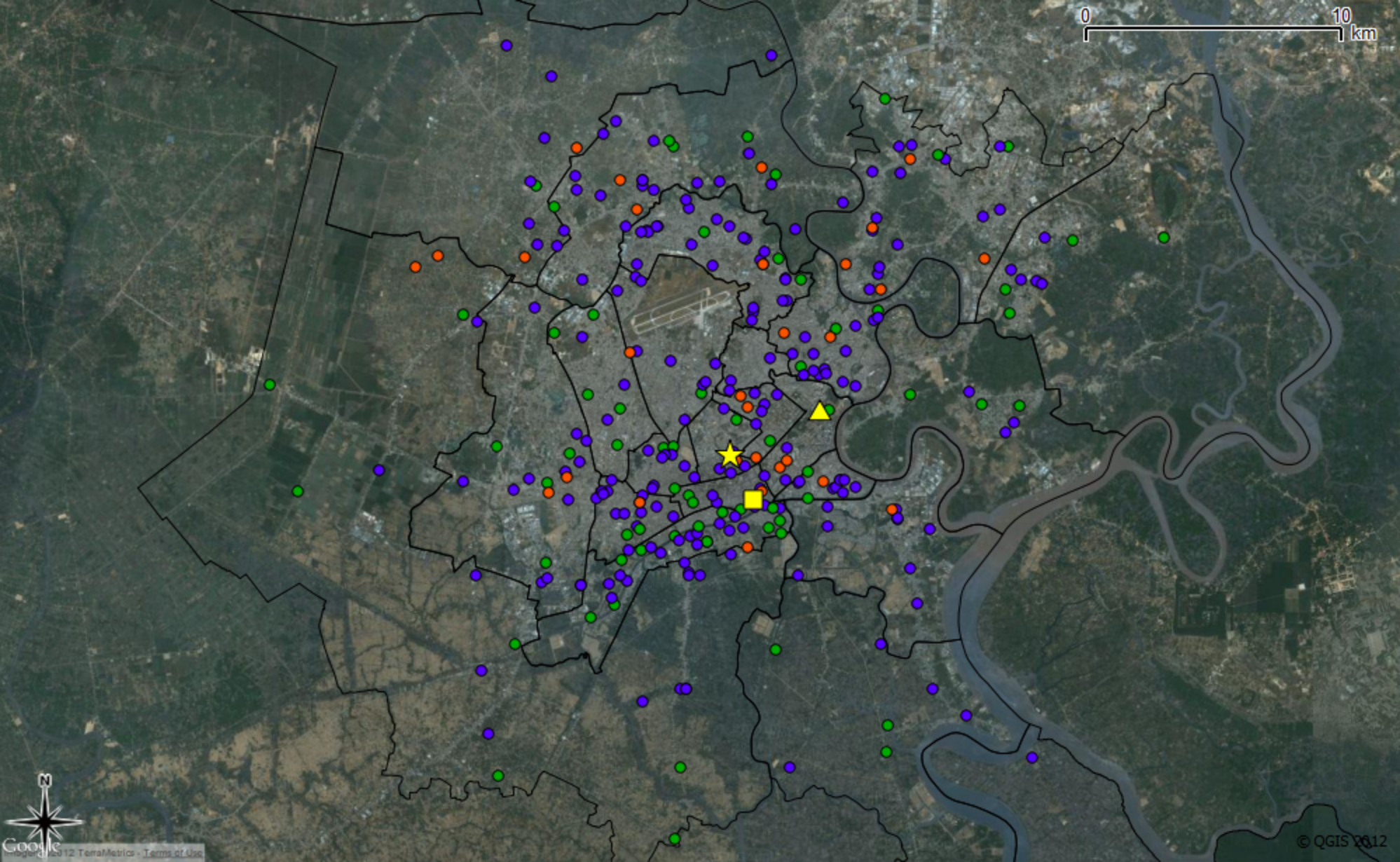

0 10 km

Supplement: Supplementary data 1 — Supplementary material. [file mmc1.pdf]
